# Personality, capability, and context: a 30 year integrative review and theoretical expansion of the Big Five framework

**DOI:** 10.3389/fpsyg.2026.1766680

**Published:** 2026-03-24

**Authors:** Abdulelah Alnafisah

**Affiliations:** Business Administration Department, Saudi Electronic University, Riyadh, Saudi Arabia

**Keywords:** Big Five framework, Big Five model, Big Five traits, integrative review, trait-capability-context (TCC) model

## Abstract

**Introduction:**

Personality assessments based on the Big Five framework remain widely used in employee selection and organizational research. However, debates persist regarding their predictive strength and practical relevance across dynamic workplace contexts. This study revisits these debates by examining how personality traits interact with individual capabilities and contextual factors in predicting job performance.

**Methods:**

This integrative review followed PRISMA guidelines and analyzed peer-reviewed studies published between 1991 and 2024. Literature was searched across Web of Science, Scopus, ScienceDirect, and the Saudi Digital Library. A total of 315 records were identified, 125 duplicates were removed, and 190 studies were screened. After the eligibility assessment, 43 studies met the inclusion criteria and were analyzed using descriptive synthesis, thematic coding, and alignment with an integrative framework.

**Results:**

The review found that conscientiousness consistently emerged as the strongest predictor of job performance across occupations. Other traits, such as extraversion, openness, agreeableness, and neuroticism, showed more context-dependent relationships with performance outcomes. Across the analyzed studies, three major themes emerged: (1) personality traits as behavioral resources, (2) capabilities such as learning agility, adaptability, and job crafting as mechanisms translating traits into behavior, and (3) contextual moderators including job design, leadership climate, and cultural environment.

**Discussion:**

The study proposes the Trait–Capability–Context (TCC) model, which explains how personality traits interact with individual capabilities and organizational environments to influence performance outcomes. The model suggests that traits alone are insufficient predictors of job performance and must be considered alongside capability development and contextual conditions. This integrative framework provides a more realistic foundation for employee selection and talent development, highlighting the need for future research using longitudinal and multi-level designs that model how traits, capabilities, and contexts interact dynamically over time, across careers, and under dynamic conditions.

## 1 Introduction

Organizations increasingly rely on personality measures to predict workplace behavior and job performance (Biea et al., 2023). The Big Five Model, conscientiousness, extraversion, openness, agreeableness, and neuroticism has been the dominant framework in this research domain for more than three decades (Murphy, 2005; Huo and Jiang, 2023; Furnham and Cheng, 2024; Laguía et al., 2024; Lee et al., 2024). Meta-analytic evidence demonstrates that conscientiousness consistently predicts performance across occupations, while other traits show more role-dependent associations, for example, extraversion in sales and neuroticism in stress-prone roles (Zell and Lesick, 2022).

However, traditional personality research assumes traits are stable, context-independent predictors. This view aligns with earlier organizational environments where job tasks were relatively stable, work contexts were predictable, and performance was largely driven by compliance and cognitive skills (Barrick and Mount, 1991; Penney et al., 2011). Contemporary workplaces, by contrast, are characterized by technological disruption, dynamic role expectations, hybrid work arrangements, and continuous upskilling demands (Pletzer and Abrahams, 2025). These transformations challenge trait-only perspectives and call for an updated theoretical synthesis (He et al., 2019; Zell and Lesick, 2022; Alderotti et al., 2023).

Although earlier reviews have debated the limited predictive validity of Big Five Tests (Nguyen et al., 2021; Angelini, 2023; Van Iddekinge et al., 2023), these debates rarely explain why and under what conditions validity fluctuates. To address this gap, the present review introduces a new integrative framework, the Trait- Capability-Context Model, derived from the synthesis of 43 studies. The TCC model integrates the Resource-based view (Wernerfelt, 1995) and Dynamic Capability Theory (Teece and Pisano, 1994), revealing that performance emerges not only from traits but also from capabilities (adaptability, learning agility, job crafting) and contextual conditions (job design, cultural environment, leadership climate).

This review contributes to the field in the following ways. First, it offers a contemporary synthesis that moves beyond static trait prediction, clarifying the inconsistent results reported across industries, job roles, and methodological designs. Second, it presents a novel integrative theoretical model that unifies the trait, capability, and context perspectives. Third, it outlines future research pathways that reflect the realities of technologically mediated, continuously evolving workplaces.

### 1.1 Historical developments of Big Five personality tests

The Big Five Personality Tests originated in the 1940s–1960s when Psychologists like Raymond Cattell (Cattell, 1957) identified key personality traits using data analysis. This marked the foundation of the Big Five model. In the 1980s, Costa and McCrae introduced the NEO Personality Inventory (Costa and McCrae, 1980). This led to the wide acceptance of the Big Five Test, making it easy to measure. In the 1990s, the model gained popularity for predicting job performance. Organizations started using it for hiring decisions and building effective teams. In the 2000s, studies explored its use globally, revealed cultural biases, and prompted efforts to adapt tests for different contexts (Hurtz and Donovan, 2000). In the 2010s, digital tools enhanced how personality traits are assessed (Dan et al., 2021).

### 1.2 The five-factor model of personality

Horstmann and Ziegler (2016) defined personality as how an individual responds to external stimuli in a stable, consistent manner. Personality traits have been used in psychology research as predictors of human behavior (Andreassi, 2010; Luo et al., 2023a; Harms et al., 2024) and how behavior can be formed (Babaei et al., 2018; Luo et al., 2023b; Adler, 2024). Researchers widely use the Big Five Personality traits, a model that distinguishes personality characteristics into five major dimensions: conscientiousness, openness to experience, extraversion, agreeableness, and neuroticism (McCrae and Costa, 1987; Goldberg, 2001; El Bahri et al., 2023).

Conscientiousness refers to a person’s degree of organization, responsibility, self-discipline, and goal-directed behavior (Colbert et al., 2004). It is consistently the strongest predictor of job performance across occupations, largely due to its influence on dependability, persistence, and attention to detail. Employees high in conscientiousness tend to be more reliable, productive, and better at planning and prioritizing tasks (Beitelspacher and Getchell, 2023). Neuroticism or emotional instability measures psychological problems, such as anxiety, emotional reactivity, and mood instability (Rezaiee Fard and Amiri, 2024). In most work settings, lower levels of neuroticism are associated with better job performance, particularly in stressful environments. High neuroticism may lead to decreased resilience, poor coping strategies, and impaired decision-making under stress (Sauer-Zavala and Barlow, 2021).

Extraversion refers to the extent to which individuals are outgoing, energetic, and sociable. Extraverts often thrive in roles that require communication, persuasion, and leadership (Walker, 2020). The trait has shown moderate predictive validity in performance for jobs that involve teamwork or external engagement but is less critical in solitary or technical roles (Lan et al., 2021). The openness to experience reflects intellectual curiosity, creativity, imagination, and a preference for novelty and variety, and is increasingly valued in dynamic work environments (Hung, 2020). Individuals high on openness are often better suited for roles that demand innovation, adaptability, and complex problem-solving, such as research and development, design, or strategic planning (Zheng et al., 2024).

Agreeableness captures traits such as cooperation, trustworthiness, altruism, and empathy (Kao and Chiou, 2020). In workplace contexts, it is often associated with interpersonal harmony, conflict avoidance, and teamwork. High agreeableness can enhance collaboration and reduce workplace tension, and its predictive strength is role dependent (Shiwen and Ahn, 2023).

### 1.3 From traits to capabilities

Scholars increasingly acknowledge that enduring traits alone cannot account for adaptive performance in modern organizations (He et al., 2019; Pletzer and Abrahams, 2025). Accordingly, constructs such as learning agility, adaptability, and job crafting have emerged as self-regulatory capabilities that enable individuals to deploy their personality traits effectively across changing contexts. Learning agility refers to the willingness and ability to learn from experience and apply that learning to novel challenges (Lombardo and Eichinger, 2000; Milani et al., 2021). Researchers advanced this concept by identifying dimensions such as feedback seeking, reflection, and cognitive flexibility that transform latent personality potential into observable learning behavior (DeRue et al., 2012). Empirical studies demonstrate that learning agility facilitates leader development and talent identification, often serving as a mediator between traits and leadership success (Finkelstein et al., 2018; De Meuse and Harvey, 2022).

Adaptability complements learning agility by capturing behavioral readiness to adjust to evolving demands, uncertainty, or environmental change (Wilmot and Ones, 2021). It operationalizes trait flexibility, especially openness to experience and emotional stability, within real-world performance. A third construct, job crafting, extends the adaptability paradigm by describing how employees proactively alter the boundaries of their tasks, relationships, or cognitive framing of work to achieve a better fit and meaning (Wrzesniewski and Dutton, 2001). Job crafting links personality-driven motives, traits such as conscientiousness, openness, and extroversion, to proactive voluntary design behaviors that enhance engagement and performance (Petrou et al., 2012).

## 2 Method

An integrative review methodology has been employed, guided by established protocols for synthesizing empirical and theoretical literature. An integrative review systematically evaluates published publications within a specified domain (Paul et al., 2021). Its objectives include defining concepts, reviewing theories and evidence, systematically analyzing issues, and providing a comprehensive overview of the topic (Doolen, 2017). The integrative review compiles and assesses conclusions from various studies and determines implications regarding the manner and rationale for further research on the subject (Cronin and George, 2023). This study adhered to the Preferred Reporting Items for Systematic Reviews and Meta-Analyses (PRISMA) guidelines to ensure transparency in the selection, inclusion, and exclusion of studies (Liberati et al., 2009). The process involved literature search and identification, screening and eligibility assessment, critical appraisal, and synthesis and interpretation.

### 2.1 Review objectives and questions

RQ1: How do Big Five traits predict job performance across contemporary contexts?

RQ2: What roles do individual capabilities (e.g., adaptability, learning agility, job crafting) play in shaping the relevance and predictive value of Big Five traits?

RQ3: How do contextual factors (job complexity, organizational support, cultural environment) interact with personality traits to influence employee selection outcomes?

RQ4: How can the Trait–Capability–Context (TCC) Model provide a more complete explanation of performance prediction than traditional trait-only approaches?

### 2.2 Search strategy

The initial search terms were personality traits, personal characteristics, personality test, Big Five Personality traits, Big Five Personality test, the relationship between personality traits and job performance, and the relationship between Big Five Personality traits and job performance. Articles published in English and indexed in Web of Science, Scopus, ScienceDirect, and Saudi Digital Library were shortlisted. They are chosen for their extensive coverage of peer-reviewed psychology, management, and organizational behavior publications. The search covered the period from January 1991 to December 2024, corresponding to the period following the widespread adoption of the Big Five model in organizational research. Full copies of identified journal articles that met the inclusion criteria, based on title, abstract, and subject overview, were obtained for data synthesis. Journal articles determined through reference lists and bibliographic searches were also considered based on titles.

### 2.3 Eligibility criteria

Table 1 presents the inclusion and exclusion criteria that were published *a priori* to ensure consistency and relevance.

**Table 1:** Parameters of the inclusion and exclusion criteria.

Inclusion Criteria	Exclusion Criteria
Studies using Big Five personality traits to explain job performance, either through empirical data or theoretical models.	Studies without experimental or theoretical relevance to the Big Five Model or job performance.
Studies evaluating the relationship between Big Five Personality traits and job performance across various job roles and industries. Studies presenting conceptual models or theoretical frameworks related to personality and work behavior.	Studies that do not focus on employees or workplace performance outcomes. Opinion pieces, non-peer-reviewed articles, and editorials.
Empirical studies reporting measurable outcomes grounded in recognized personality or HRM theories. Studies published from 1950 onwards, aligning with the historical development of the Big Five Model. Studies published in peer-reviewed journals.	Articles lacking adequate methodological transparency. Duplicated studies across databases. Research papers in a language other than English, necessary to ensure consistency in interpretation and quality appraisal.

### 2.4 Study selection process

**Figure 1:**
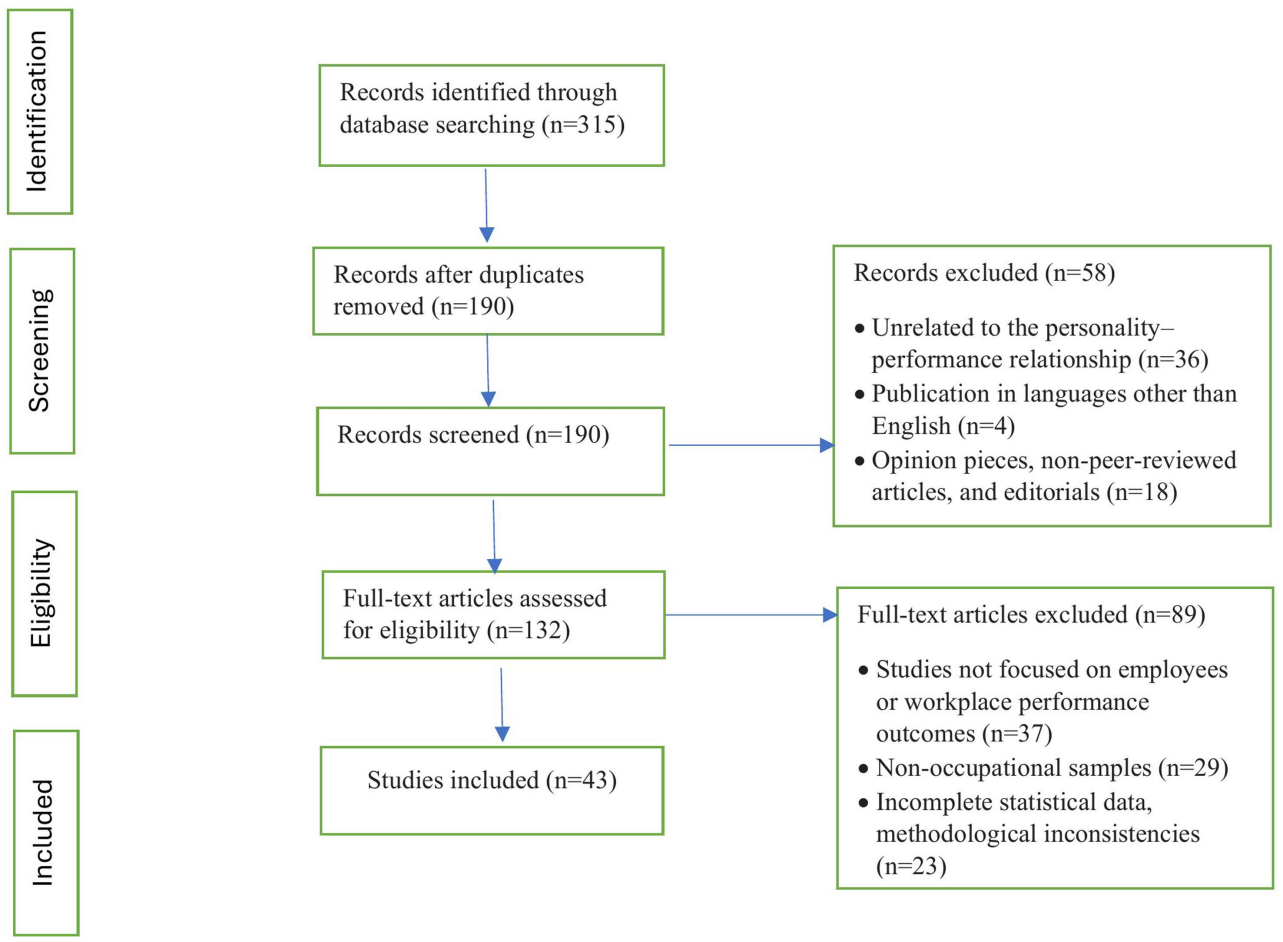
PRISMA diagram.

A total of 315 studies were identified. After removing 125 duplicates, 190 studies were screened for relevance. Of these, 132 full-text articles were assessed against the inclusion criteria. Finally, 43 studies that met all the requirements were included in the review (Figure 1 for the PRISMA diagram).

### 2.5 Critical appraisal and data extraction

Data extraction employed a standardized matrix capturing: Author(s), year, country, and publication outlet, Research design (cross-sectional, longitudinal, meta-analytic, or mixed-method), Personality measures used (e.g., NEO-PI-R, BFI, TIPI), Mediating/moderating constructs (e.g., learning agility, adaptability, job crafting), Sample size and industry context, Main findings and limitations.

To ensure the reliability of findings, a structured quality assessment was conducted for all included studies. Empirical studies were evaluated using the JBI Critical Appraisal Checklist for Analytical Cross-Sectional Studies (Barker et al., 2023). Theoretical works were assessed using a conceptual appraisal rubric focused on coherence, theoretical alignment, and relevance. Appraisal results were used to inform both inclusion decisions and the interpretive weight assigned during synthesis (Hong and Pluye, 2019).

Critically, these appraisal outcomes influenced synthesis weighting: studies with stronger designs and higher quality ratings were given proportionally greater interpretive emphasis during thematic integration. This ensured that the conclusions of the review reflected both evidence breadth and methodological robustness.

### 2.6 Synthesis and integration

Following the Prisma framework, data synthesis followed a three-step procedure: descriptive analysis, thematic coding, and integrative framework alignment (Hopia et al., 2016; Younas et al., 2022). Descriptive analysis details resource distribution, design patterns, and sample traits, including year, journal, location, methods, size, industry, and study type, focusing on personality tests and results. Thematic coding linked traits to performance through mediating capabilities. Key recurring topics, like conscientiousness improving job performance, emerged (Abou-Shouk et al., 2022; Shiwen and Ahn, 2023). Similar ideas were grouped into broader categories, like personality traits predicting job performance, capabilities, and contextualized personality tests. Key themes included traits predicting success across industries, their effect on learning new skills, and the workplace context’s role in personality-based hiring. The integrative framework alignment maps findings onto the Trait-Capability-Context model, whose structure shows how dispositional, self-regulatory, and contextual factors determine job performance.

## 3 Results

The final data set includes 43 peer-reviewed studies published between 1991 and 2024 across 17 journals. The study types include nine meta-analyses, 26 quantitative approaches (cross-sectional/field/survey), five experimental or longitudinal designs, and three mixed methods/qualitative studies. Geographically, the studies cover 16 countries across five continents, including the US, Europe, Asia, Africa, and multi-country global samples. This diversity highlights the Big Five’s worldwide relevance and emphasizes the contextual differences that influenced thematic variations.

Personality research has shown a steady increase since 2010, with a big jump in studies published between 2019 and 2024 (Ones et al., 2025). Studies from 2010 to 2015 focused on how personality traits like conscientiousness could predict job performance (Blickle et al., 2013). But in recent years, researchers have explored new areas, such as linking personality with emotional intelligence (Di Fabio and Saklofske, 2021), remote work conditions, and virtual teams (Flavián et al., 2022) and problems in diverse workforces (Van Iddekinge et al., 2023).

Moreover, earlier studies looked at personality in general industries (Dong and Dumas, 2020). However, recent studies have focused on specific industries, such as healthcare, where studies have used personality tests to predict burnout and patient interaction skills (Handayani and Kuntarti, 2022), technology where the focus is more on skills and adaptability (Lu et al., 2023) and marketing, where personality traits like extraversion and agreeableness are still linked to success (Patel et al., 2024). In contrast, more long-term studies have recently been conducted to see how personality traits affect performance over time (den Hartog et al., 2020; Iliescu et al., 2023; Kordsmeyer et al., 2024). For example, Zheng et al. (2024) identified that conscientiousness was helpful early on, but traits like openness to new experiences were more important for long-term growth and innovation.

**Figure 2:**
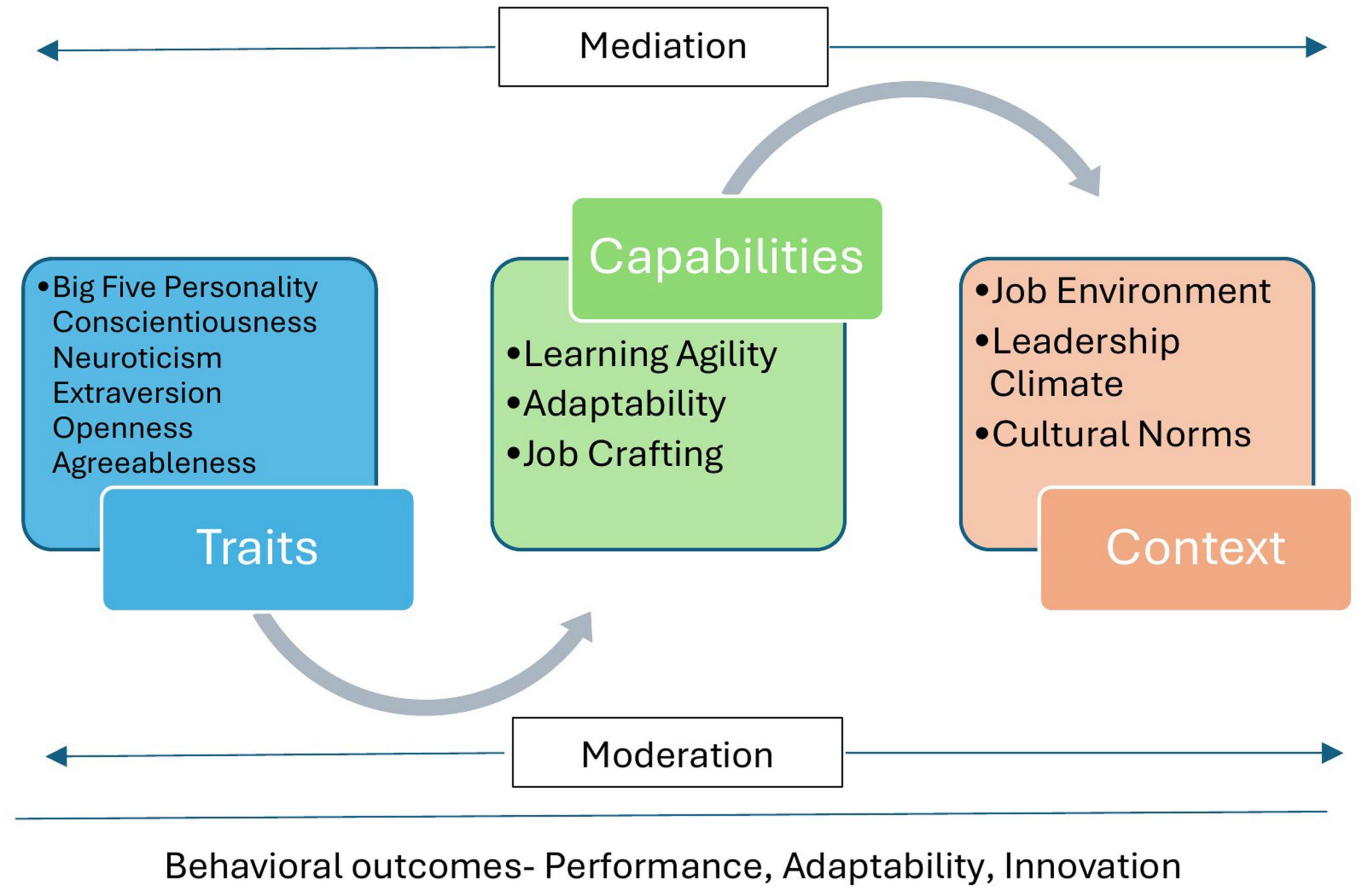
Trait–capability–context (TCC) model.

Three overarching themes emerged from the synthesis, aligning with the proposed Trait–Capability–Context (TCC) Model. The model draws conceptually on the Resource-based View (Wernerfelt, 1995) and Dynamic Capability Theory (Teece and Pisano, 1994), which views human characteristics as strategic resources that must be mobilized through learning and adaptation to generate value. In this view, traits represent enduring psychological resources that shape behavioral potential, such as conscientiousness, fostering persistence, and openness, enabling exploration. Capabilities serve as translational mechanisms that activate and channel trait potential towards effective behavior, such as learning agility, adaptability, and job crafting. Context, comprising organizational culture, leadership climate, task structure, and cultural norms, moderates both the expression of traits and the development of capabilities. The TCC model posits that performance emerges from the interaction of these three components rather than from traits alone, providing a cohesive framework that explains how who people are (traits) interact with what they can do (capability) and where they work (context). Figure 2 illustrates these relationships. Each theme and its subthemes are discussed below.

### 3.1 Theme 1: trait

#### 3.1.1 Sub-theme: conscientiousness as the strongest predictor (18 studies)

Conscientiousness emerged as the most consistent and robust predictor of job performance (Vinchur et al., 1998; Alessandri and Vecchione, 2012). Over 80% of the included empirical studies reported statistically significant positive associations between conscientiousness and performance outcomes, including task completion, reliability, and goal orientation (van Aarde et al., 2017). Meta-analytic findings also support its validity, demonstrating that effect sizes vary depending on the occupational context. In roles with high task interdependence, such as team-based healthcare or collaborative R&D environments, conscientiousness alone was less predictive, suggesting potential interaction effects with traits like agreeableness or neuroticism (Vergauwe et al., 2022; Zell and Lesick, 2022).

### 3.2 Theme 2: capability

#### 3.2.1 Sub-theme: adaptability, learning agility, job crafting as mediators and moderators (21 studies)

The review identified key mediators such as commitment (Matzler and Renzl, 2007), motivation (Barrick et al., 2001), job requirements (Tett and Burnett, 2003), job characteristics (Hung, 2020), job crafting behavior (Geldenhuys and Peral, 2020), emotional intelligence (Murmu and Neelam, 2022) and adaptability (Wilmot and Ones, 2021; Schermer et al., 2024) influencing traits and job performance relationship. A consistent pattern shows that adaptability and learning agility strengthen the link between personality and performance (Blickle et al., 2013; Fichter et al., 2020; Hung, 2020). For example, employees low in openness but high in adaptability achieved equivalent performance outcomes. Also, researchers demonstrated that learning-oriented behavior mediates conscientiousness-performance relationships (Penney et al., 2011; Rashid et al., 2016).

Studies reveal that job crafting, an employee-driven task motivation, is a mechanism that translates personality potential into observable performance. Similarly, researchers demonstrated that self-regulation and intrinsic motivation mediate the influence of conscientiousness and openness on work outcomes (Geldenhuys and Peral, 2020). The author identified balanced extraversion as the optimal profile for adaptive performance in sales (Grant, 2013). Together, these findings emphasize self-regulatory activation as the link between traits and behavior (Peterson et al., 2009). This supports the capability component of the TCC framework, showing that dynamic competencies activate the behavioral expression of traits.

### 3.3 Theme 3: context

#### 3.3.1 Sub-theme: trait-role dependency and situational variance (23 studies)

The overall practical utility of personality tests varies across roles, industries, and cultures (Thoresen et al., 2004; He et al., 2019). This highlights the importance of considering trait-job fit rather than applying personality tests universally (Blickle et al., 2013). Findings show that the predictive power of traits such as neuroticism, extraversion, openness, and agreeableness depends on occupational context and role demands. For example, Neuroticism consistently shows a negative relationship with job performance across multiple studies (Tett et al., 1991). Low neuroticism was linked to better stress management, workplace adaptability, and decision-making under pressure. This trend held across industries, particularly in high-pressure environments such as law, healthcare, and leadership (Masood et al., 2017).

Extraversion showed a domain-specific association with job performance (Hunthausen et al., 2003). It was positively associated with success in sales, leadership, and customer service roles, where interpersonal engagement and assertiveness are valued (Grant, 2013; Wilmot et al., 2019). However, in fields like IT and accounting, the trait had little or no predictive power. Studies highlighted the importance of role-personality fit in moderating extroversion’s relevance to job outcomes (Sartori et al., 2017; Lan et al., 2021).

The relationship between openness and job performance was found to be inconsistent (Ayub et al., 2017). While some studies found a moderate link between openness and creativity, innovation, and learning adaptability, other studies reported negligible correlations in structured routine jobs. This suggests that the utility of transparency may be role-specific and mediated by job complexity and change orientation (Abou-Shouk et al., 2022). Agreeableness demonstrated weak to moderate associations with performance, primarily in roles emphasizing teamwork, cooperation, and conflict resolution (Shiwen and Ahn, 2023). In customer-facing roles and care-driven professions, higher agreeableness was associated with better relational outcomes and peer evaluations. However, in high competition or assertive negotiation contexts, high agreeableness was not consistently advantageous (Chandrasekara, 2019).

Cultural context emerged as a critical moderator of trait expression and test validity (Penney et al., 2011; Deinert et al., 2015). Studies conducted in collectivist cultures, including South Korea, China, and Pakistan, showed attenuated trait-performance correlations compared with a Western, individualistic context (Rashid et al., 2016; Lan et al., 2021; Han et al., 2024). In a collectivist environment, social harmony and group alignment often shape behavioral norms, leading to reduced differentiation in trait-based behavior and performance ratings. For example, extraversion and openness predicted leadership and innovation more strongly in US and European samples, while agreeableness and conscientiousness were valued and predictive in Asian and Middle Eastern contexts, emphasizing cooperation and role conformity (Ghani et al., 2016; Abou-Shouk et al., 2022).

Additionally, concerns remain about response distortion in self-report inventories, particularly in high-stakes settings (Sjöberg, 2015; Jeong et al., 2017). Though newer testing formats reduce faking, their accuracy still falls short when used in isolation (Lopez et al., 2019; Purpura et al., 2022). Table 2 summarizes the 43 studies analyzed, organized chronologically to illustrate the evolution of research on the Big Five and job performance from static trait models to dynamic, context-sensitive frameworks.

**Table 2:** Comparative overview of included studies.

Authors	Journal	Country/Industry	Sample size	Study design	Personality measure	Additional constructs	Findings
Barrick and Mount (1991)	Personality and Individual Differences	USA/Mixed industries	117 studies	Meta analysis	Big Five inventories	Cognitive ability	Conscientiousness most consistently predicts job performance.
Vinchur et al. (1998)	Journal of Applied Psychology	USA /Sales	55 samples	Meta analysis	Big Five	Cognitive ability	Big Five traits and cognitive ability predict sales performance.
Barrick et al. (2001)	International Journal of Selection and Assessment	USA / Organizational	Review	Review	—	—	Big Five research consolidated for early 2000s insights.
Hunthausen et al. (2003)	Journal of Applied Psychology	USA / Business	280	Field study	NEO-PI-R	Frame of reference	The frame of reference improves personality test validity.
Tett and Burnett (2003)	Human Performance	USA / Conceptual	—	Qualitative/Conceptual	—	Situational strength	Personality–situation interactions explain job performance.
Thoresen et al. (2004)	Journal of Applied Psychology	USA/Corporate	450	Quantitative/Longitudinal	NEO-PI-R	Job satisfaction	Big Five traits affect job performance at time stages.
Matzler and Renzl (2007)	International Journal of Human Resource Management	Austria/Knowledge sector	210	Quantitative/Survey	NEO-PI-R	Affective commitment	Personality traits predict affective commitment and knowledge sharing.
Peterson et al. (2009)	International Journal of Selection and Assessment	USA / Recruitment	515	Quantitative/Survey	Big Five inventories	Faking behavior	Personality test fakers are often still hired.
Converse et al. (2009)	Human Performance	USA / Organizational	280	Experimental	NEO-PI-R	Faking	Faking reduces multiple predictor model accuracy.
Penney et al. (2011)	Human Performance	USA/Review	—	Review	—	Context factors	Context, boundaries, and situational factors moderate personality-performance links.
Alessandri and Vecchione (2012)	European Journal of Psychological Assessment	Italy/Organizational	340	Quantitative/Survey	Big Five inventories	Higher-order traits	Higher-order Big Five factors better predict job outcomes.
Grant (2013)	Psychological Science	USA/Sales	340	Experimental	Big Five inventories	Ambiversion	Ambiverts outperform extroverts/introverts in sales.
Blickle et al. (2013)	Applied Psychology	Germany/Business	400	Quantitative/Survey	Big Five inventories	Political skill	Political skill enhances the impact of personality traits on performance.
Judge and Zapata (2014)	Personnel Psychology	USA/Organizational	302	Experimental	NEO-PI-R	Situation strength	Situation strength influences Big Five’s predictive power.
Sjöberg (2015)	Scandinavian Journal of Psychology	Sweden/Testing	160	Quantitative	Big Five inventories	Faking detection	Statistical correction improves faking detection in tests.
Deinert et al. (2015)	Leadership & Organization Development Journal	Germany/Corporate	275	Quantitative/Survey	Big Five inventories	Transformational leadership	Transformational leadership subtypes linked to Big Five traits.
Rashid et al. (2016)	Journal of Business Studies Quarterly	Pakistan/Banking	280	Quantitative/Survey	Big Five inventories	—	Personality predicts frontline banking staff performance.
Ghani et al. (2016)	Asian Social Science	Malaysia/Public sector	310	Quantitative/Survey	Big Five inventories	Leadership	Public sector leaders’ traits influence subordinates’ performance.
Masood et al. (2017)	Pakistan Journal of Psychology	Pakistan/Police	200	Quantitative/Survey	Big Five inventories	—	Conscientiousness, extraversion, and agreeableness predict police performance.
Sartori et al. (2017)	Journal of Work and Organizational Psychology	Italy, Spain/Manufacturing	250	Mixed method	Big Five inventories	Work perception	Workers’ perception highlights complex personality-performance links.
Jeong et al. (2017)	Journal of Business and Psychology	South Korea/Corporate	320	Experimental	Big Five inventories	Faking	Faking reduces the validity of personality assessments.
Ayub et al. (2017)	International Journal of Business & Management	Pakistan/Business	230	Quantitative/Survey	Big Five inventories	Conflict styles	Big Five and conflict styles predict performance.
van Aarde et al. (2017)	South African Journal of Industrial Psychology	South Africa/Multiple	90 studies	Meta-analysis	Big Five	—	Validity of Big Five traits confirmed in the South African context.
Wilmot et al. (2019)	Journal of Organizational Behavior	Global/Leadership and sales	50 studies	Meta-analysis	Big Five	Role type	Extraversion positively predicts performance in leadership/sales.
Chandrasekara (2019)	Asian Journal of Multidisciplinary Studies	Sri Lanka/Education	215	Quantitative/Survey	BFI	Job satisfaction	Big Five predicts teacher job satisfaction and performance.
Lopez et al. (2019)	Journal of Applied Psychology	USA/Testing	300	Experimental	BFI	Faking	New warning procedures reduce faking in tests.
He et al. (2019)	Journal of Vocational Behavior	China/Multiple	130 studies	Meta-analysis	Big Five	—	Conscientiousness and extraversion are the most predictive traits.
Fichter et al. (2020)	Heliyon	Germany, Austria, Switzerland/Startups	412 founders	Quantitative/Survey	Big Five inventories	Stress	Perceived stress is related to the founders’ personality (emotional stability negative, conscientiousness positive), and in turn hurts performance.
Hung (2020)	Asian Academy of Management Journal	Taiwan/Business	285	Quantitative/Survey	Big Five inventories	Work effort and learning	“Working smart” and “working hard” mediate personality–performance link.
Geldenhuys and Peral (2020)	South African Journal of Psychology	South Africa/Services	289	Quantitative/Survey	Big Five inventories	Job crafting	Job crafting mediates personality–performance relationships.
den Hartog et al. (2020)	Small Group Research	Netherlands /Teams	215	Quantitative/Survey	Big Five inventories	Team innovation	Personality trait variance in self-managed teams is positively associated with team innovation.
Lan et al. (2021)	Tourism Management Perspectives	China / Hospitality	260	Quantitative/ Survey	Big Five inventories	Job satisfaction	Personality affects job satisfaction and performance in hospitality.
Wilmot and Ones (2021)	Psychological Bulletin	USA/Global	Meta-analysis	Meta-analysis	Big Five	Job characteristics	Job characteristics moderate personality-performance links.
Zell and Lesick (2022)	Journal of Vocational Behavior	Global/Multiple	50 + meta-analyses	Meta-analysis	Big Five	—	Synthesizes 50 + meta-analyses; confirms consistent Big Five-performance links.
Murmu and Neelam (2022)	Asian Journal of Management	India/Virtual teams	235	Quantitative/Survey	Big Five inventories	Emotional intelligence	Emotional intelligence and Big Five jointly impact virtual team performance.
Purpura et al. (2022)	Personality and Individual Differences	USA/Testing	200	Quantitative	Big Five inventories	TF-IDF detection	TF-IDF method detects fake answers in personality scales.
Vergauwe et al. (2022)	European Journal of Work and Organizational Psychology	Belgium/Business	210	Quantitative/Survey	Big Five inventories	Work passion	Passion for work tied to general and maladaptive traits.
Soomro et al. (2022)	Leadership in Education	Pakistan/Education	300	Quantitative/Survey	Big Five inventories	Organizational cynicism	Organizational cynicism mediates personality-performance link.
Abou-Shouk et al. (2022)	Employee Relations	Egypt, Jordan / Tourism	355	Quantitative/Survey	Big Five inventories	Knowledge sharing	Personality and knowledge sharing predict innovation.
Shiwen and Ahn (2023)	International Journal of Hospitality Management	South Korea/Hospitality	260	Quantitative/Survey	Big Five inventories	—	Extraversion and agreeableness enhance restaurant staff performance.
Schermer et al. (2024)	Journal of Intelligence	Canada/Conceptual	—	Review	—	Intelligence	Revisits links between intelligence and Big Five.
Lee et al. (2024)	Journal of Applied Psychology	USA/Organizational	100 studies	Meta-analysis	Big Five	Psychological contract breach	Big Five relates to psychological contract breach and performance.
Han et al. (2024)	Group and Organization Management	South Korea/Teams	27 studies	Meta-analysis	Big Five	Team composition	Aggregate team personality traits affect group performance.

## 4 Discussion

The findings of this integrative review confirm that the Big Five personality model is valuable for understanding personality traits and predicting general behavior patterns (Dimitriou and Galanakis, 2022; Kostiani and Galanakis, 2022). It allows organizations to assess traits related to employee performance, such as conscientiousness, contributing to a better understanding of how individuals may fit into specific job roles. The consistent predictive value of conscientiousness across the reviewed studies supports the Resource-Based View (Wernerfelt, 1995), showing that certain personality traits can function as valuable and enduring internal resources. In roles that prioritize dependability, self-regulation, and task commitment, conscientiousness appears to provide organizations with a stable foundation for performance, thereby reinforcing its strategic value as a human asset (Luo et al., 2023b).

However, this review also underscores that the predictive strength of personality traits is neither uniform nor context-free (Furnham, 2002). The Big Five, while useful, are insufficient as standalone predictors of performance (Tett et al., 1991; Anvari et al., 2023). However, the deeper value of this review lies in explaining why this is the case and in proposing a new integrative model that advances the field. The TCC model offers a comprehensive explanation that performance emerges from the interplay of traits and capabilities and contextual conditions. Conscientiousness predicts performance consistently, but its effect strengthens when supported by capabilities like self-regulation and situational resources like autonomy (Furnham, 2012; Nehra et al., 2022). Conversely, personality traits such as openness or extraversion fluctuate in predictive value depending on job complexity, team demands, or leadership style (Fetvadjiev and van de Vijver, 2015). The review also expands personality research into the future of world domains. Digital transformation, hybrid work, and rapid reskilling intensify the relevance of capabilities such as adaptability, agility, and emotional intelligence, which operate as trait activators. Thus, personality prediction becomes more accurate when embedded in dynamic capability and contextual models (Pletzer and Abrahams, 2025).

This supports the Dynamic Capability Theory (Teece and Pisano, 1994) perspective, which emphasizes the importance of cultivating individual capabilities that allow employees to respond effectively to change. Traits such as openness to experience were found to be more relevant in these contexts, but only when combined with evidence of an individual’s potential for growth and learning (Judge and Zapata, 2014; Murmu and Neelam, 2022). Hence, the review suggests that while stable traits may serve as foundational indicators of role fit and reliability based on the Resource-Based View (Wernerfelt, 1995), sustainable performance in modern workplaces increasingly depends on an individual’s capacity to adapt and evolve, as emphasized by Dynamic Capability Theory (Di Stasio and van de Werfhorst, 2016).

### 4.1 Recommendations for refining Big Five assessments: toward greater precision

The results have shown that certain advancements can make the Big Five tests more precise, useful, and inclusive. The first future scenario concerns context-based testing (Sesini et al., 2024). Administering personality tests multiple times across different situations can improve accuracy by capturing how traits manifest under varying work conditions, such as stress, teamwork, or autonomy. However, full-scale repeated testing is often impractical in recruitment due to time and resource constraints. A feasible approach is a modular or phased assessment where an initial Big Five test is supplemented with short, context-specific follow-ups during onboarding or training. This maintains predictive validity while ensuring efficiency and sustainability.

The second advancement is adding personal growth tools, where tests may offer personalized advice for self-improvement and career growth. Concerning this, work difficulties can be resolved by job crafting according to their characteristics (Kooij et al., 2015; Oldham and Fried, 2016). Job crafting refers to proactive actions by employees to redesign their work, such as changing job characteristics to align with their personal preferences, needs, goals, and skills (Tims et al., 2013; Turek et al., 2024). Job crafting can take three forms: (1) Task crafting, where employees are allowed to alter the type, characteristics, and number of tasks, such as creating new ideas or modifying the way of doing the work; (2) Relationship crafting, when employees are allowed to change the people they interact with and how to interact in their job; (3) Cognitive crafting, when employees are enabled to change their perspective towards their tasks or redefine the aims of their job, which positively impact their performance (Wang et al., 2024).

Job crafting lets employees modify their roles to fit their aspirations, which can enhance performance and satisfaction (Demerouti et al., 2024; Saleem et al., 2024). The third advancement is incorporating cultural adaptations, where versions tailored to different cultures would address global applicability (Thalmayer et al., 2022). The fourth is about using AI enhancements to make assessments more accurate and efficient (Pellert et al., 2024; Pletzer and Abrahams, 2025). The fifth one includes broader assessments where tests can combine personality with emotional and social intelligence for a comprehensive picture (Irwing et al., 2024).

### 4.2 Theoretical and practical implications

This review advances personality research by integrating traits, capabilities, and context in the TCC model. It shows that personality traits function as foundational dispositions, whose effects on workplace behavior are shaped by individual capabilities and contextual conditions. This perspective explains why Big Five traits, such as conscientiousness and extraversion, exhibit varying predictive power across roles, career stages, and industries. This view supports the Resource-Based View (Wernerfelt, 1995) and Dynamic Capability Theory (Teece and Pisano, 1994) in human resource management, highlighting adaptability, learning agility, and job crafting with job complexity, leadership climate, and organizational support. Moreover, this model refines existing Trait-Performance Theory to offer a more context-sensitive understanding of how personality influences performance in contemporary, technologically driven, and dynamic workplaces.

The TCC model offers several practical implications. First, the TCC framework supports the development of adaptive role architecture, where job roles are dynamically shaped around employees’ traits and capabilities rather than expecting fixed roles to fit all employees uniformly. This shifts HR from job-based management to trait-capability-context alignment, allowing the same role to be structured differently for employees with different trait-capability combinations. Second, organizations can use the TCC model to build capability-triggered personality pathways, as a diagnostic tool for understanding why high-potential employees sometimes underperform. Instead of attributing gaps to “poor fit,” organizations can analyze where the chain breaks, does the trait exist but remain dormant due to weak capability development? Or does the context suppress trait expression? This leads to tailored interventions that address the actual source of performance bottlenecks. Third, organizations can use the TCC framework to enable context engineering, where organizations redesign micro-environments such as team structures, leadership interactions, workflow designs, and digital tools to elicit desired trait expressions. For instance, structured autonomy, supportive leadership, or clear task boundaries can amplify consciousness-driven behaviors even in employees who are not naturally high on the trait.

### 4.3 Limitations and avenues for future research

While this review provides a holistic view of the relationship between Big Five personality traits and job performance, several limitations must be acknowledged. First concern is language restriction as only English language studies were included, which may limit the global representation of findings. The second concern is self-report bias, as many studies relied on self-reported personality assessments, which may be affected by social desirability or faking and may compromise the accuracy of trait measurement. Third concerns methodological variability, as cross-study comparisons are challenged by the differences in methodology, measurement tools, occupational contexts, and outcome definitions, which may limit the direct comparability and synthesis of findings.

Future research could examine how personality traits and emotional intelligence work together in leadership. It would be interesting to see how traits like extraversion and low neuroticism help leaders manage and motivate teams, which could help improve leadership training. Future research can also examine a longitudinal evaluation of Trait–Capability–Context Interactions, which will help understand how traits’ relevance evolves as employees learn, reskill, or change roles, helping organizations predict performance more accurately across career stages. Moreover, future studies can examine multi-level, multi-context, real-workflow data, ensuring findings are grounded in actual organizational settings, making them practically useful for managers. Another important area is Career-Trajectory Analysis (Personality × Capability Development) since traits may predict early career outcomes differently than mid/late career outcomes. This will show when and how traits impact success, guiding talent development, mentoring, and promotion decisions. Furthermore, future studies can examine Trait–Context alignment under technological and workplace shifts, identifying which traits help employees thrive in dynamic environments, supporting organizational resilience and strategic HR planning.

## 5 Conclusion

This review demonstrates that while Big Five personality traits contribute to understanding employee behavior, they are insufficient as standalone predictors of job performance in modern workplaces. The trait-capability-context (TCC) model developed in this review offers a comprehensive framework by showing that traits provide baseline dispositions, capability determines how those dispositions translate to performance, and context shapes when traits or capabilities matter most. Therefore, future research and practice should adopt capability-based and context-sensitive assessments, using personality tests not as gatekeeping tools but as one component within a multilayered evaluation system.

## Data Availability

The original contributions presented in the study are included in the article/supplementary material, further inquiries can be directed to the corresponding author.
